# Photobiomodulation in Promoting Cartilage Regeneration

**DOI:** 10.3390/ijms26125580

**Published:** 2025-06-11

**Authors:** Nguyen Le Thanh Hang, Ana Elena Aviña, Cheng-Jen Chang, Tzu-Sen Yang

**Affiliations:** 1 International Ph.D. Program in Biomedical Engineering, Taipei Medical University, Taipei 110, Taiwan; d845111005@tmu.edu.tw; 2 International Ph.D. Program in Medicine, College of Medicine, Taipei Medical University, Taipei 110, Taiwan; d142111012@tmu.edu.tw; 3 Department of Plastic Surgery, Taipei Medical University Hospital, Taipei 110, Taiwan; chengjen@h.tmu.edu.tw; 4 Department of Surgery, School of Medicine, College of Medicine, Taipei Medical University, Taipei 110, Taiwan; 5 Graduate Institute of Biomedical Optomechatronics, Taipei Medical University, Taipei 110, Taiwan; 6 School of Dental Technology, Taipei Medical University, Taipei 110, Taiwan; 7 Research Center of Biomedical Device, Taipei Medical University, Taipei 110, Taiwan

**Keywords:** photobiomodulation, cartilage tissue engineering, cartilage regeneration

## Abstract

Articular cartilage is an avascular and aneural connective tissue that is frequently damaged due to trauma or degenerative joint diseases, often resulting in arthritis. Its limited intrinsic capacity for self-renewal poses a significant challenge to effective repair. Hence, the development of regenerative strategies is essential to enhance the poor intrinsic healing of cartilage tissue. Photobiomodulation (PBM) has gained increasing attention as a noninvasive, drug-free, and safe approach. PBM exerts photobiological effects that promote cellular responses and reduce inflammatory conditions, all of which are beneficial for cartilage repair. Nonetheless, the efficacy of PBM varies depending on treatment parameters and treated targets. This review first summarizes PBM parameter-dependent outcomes in cartilage regeneration studies. Reported data indicate frequent use of red lasers (600–660 nm, 0–10 J/cm^2^), GaAIAs lasers (800–880 nm, 10–50 J/cm^2^), and Nd:YAG lasers (1064 nm, up to 200 J/cm^2^) in in vitro, in vivo, and clinical studies. Moreover, PBM in conjunction with cartilage tissue engineering (CTE) has shown synergistic effects, enhancing scaffold-based repair outcomes. This review additionally explores PBM applications within CTE frameworks. The summarized findings aim to inform researchers and physicians by outlining optimized PBM strategies and highlighting PBM’s strong potential in promoting cartilage regeneration, both independently and in combination with CTE.

## 1. Introduction

Articular cartilage (AC), which is a type of hyaline cartilage, is an avascular tissue located in diarthrodial joints, covering the surfaces of bones in synovial joints [1]. Its primary function is to create a smooth, lubricated joint surface and reduce load transmission with low friction to protect the underlying subchondral bone [2]. AC is recognized for its limited self-renewal capacity following damage, often leading to arthritis [3]. The two common forms of arthritis are osteoarthritis (OA) and rheumatoid arthritis (RA) [4]. Specifically, OA is characterized by the degradation of AC, formation of bone spurs, and various abnormalities in the joint and synovium, resulting in knee joint pain and morbidity due to joint wear and degradation [5]. Conversely, RA is a chronic inflammatory disease primarily targeting synovial joints, but it can also affect other organs, such as the skin, lungs, and vascular system [6]. RA can cause bone erosion and joint deformity by damaging the synovial lining leading to severe pain, inflammation, and swelling [7]. Consequently, individuals with RA may experience disability, diminished quality of life, and significant socioeconomic burdens [8].

Cartilage regeneration is most directly linked to degenerative joint conditions, particularly primary or secondary osteoarthritis and focal osteochondral defects, which form the primary focus of our review and underpin the discussion on tissue loss and structural restoration [9,10]. At the same time, photobiomodulation (PBM) has garnered increasing attention as a potential adjunctive therapy in inflammatory arthropathies. PBM may help mitigate synovial inflammation, oxidative stress, and catabolic cytokine activity, all of which contribute to cartilage degradation [11].

While our narrative synthesis did not encompass all inflammatory conditions, such as systemic lupus erythematosus, spondyloarthritis, or crystal-induced arthropathies, we included rheumatoid arthritis as a representative autoimmune model. This inclusion allows us to illustrate how PBM might influence immune-mediated inflammatory cascades that adversely affect articular cartilage [12,13]. This distinction between degenerative and inflammatory etiologies reflects the therapeutic complexity of joint disease and highlights the potential for PBM to serve as a versatile tool within a broader, multimodal treatment framework.

A commonly used traditional treatments for chronic articular cartilage defects is microfracture (MF), which aims to initiate healing of AC or osteochondral injuries by triggering an inflammatory response that leads to the formation of fibrocartilages [14]. However, MF is not suitable for defects larger than 2–4 cm^2^ [15]. Additional techniques for stimulating cartilage regeneration include the osteochondral autograft transfer system (OATS), autologous chondrocyte implantation (ACI), and autologous stem cell transplantation [15]. Each method has limitations: OATS requires advanced surgical skills and is associated with donor-site morbidity, while ACI is costly, involves long-term treatment, and requires approximately 12 months for rehabilitation [15]. These treatments have two major drawbacks. First, there is an insufficient quantity of cells available to heal damaged cartilage due to the slow migration of stem cells within the bone marrow lumen [16]. Second, there is a challenge in ensuring prolonged retention of medications or cells following in situ injection or implantation.

Photobiomodulation (PBM) has recently emerged as a promising treatment option, offering a drug-free, noninvasive, and safe alternative to conventional therapies. This review explores the potential of PBM in managing degenerative joint diseases, such as OA, by promoting cartilage regeneration, as well as its role in inflammatory arthropathies like RA through immune modulation. PBM has been shown to stimulate anti-inflammation and pain-resolving processes in various tissue and organs by enhancing biological responses, such as proliferative activity and directing cell differentiation [17]. Nonetheless, the efficacies of PBM varies significantly depending on the PBM parameters used (e.g., light sources, wavelengths, and fluences), and the specific targets treated (cells, animals, and humans).

Given this variability, the present review aims to synthesize recent findings on the effects of photobiomodulation (PBM) and tissue engineering on cartilage regeneration across multiple levels, including cellular studies, animal models, and clinical trials. A narrative synthesis was chosen and informed by a structured literature search using PubMed, Web of Science, and Scopus, encompassing studies published between 2014 and 2024. Search terms included “photobiomodulation”, “low-level laser therapy”, “cartilage regeneration”, “tissue engineering”, “osteoarthritis”, and “rheumatoid arthritis.” Peer-reviewed original research articles published in English were included, covering in vitro, in vivo, and clinical investigations that reported direct outcomes of cartilage tissue engineering (CTE) and PBM interventions on cartilage repair or joint disease models. Studies were excluded if they were not peer-reviewed, were conference abstracts, or lacked clear specification of PBM parameters such as wavelength or fluence. This inclusion strategy was adopted to accommodate the heterogeneity and evolving landscape of PBM research. Rigid systematic review criteria were deemed inappropriate due to the substantial methodological variability present across the existing body of literature.

Despite various attempts to regenerate articular cartilage following injuries, managing cartilage defects still poses clinical challenges. The integration of PBM with cartilage tissue engineering (CTE) shows promise in enhancing biological functions, thereby improving the repair of damaged or diseased tissues [18]. Nevertheless, the wide variability in three-dimensional scaffold types, light delivery systems, and irradiation protocols complicates the interpretation and standardization of findings. In light of these challenges, this review provides a comprehensive synthesis of experimental and clinical evidence. The goal is to offer insight into the therapeutic potential of PBM, both as a standalone modality and in combination with CTE, for promoting cartilage repair and regeneration.

## 2. Photobiomodulation

### 2.1. Mechanism of PBM in Promoting Cartilage Repair

**Figure 1:**
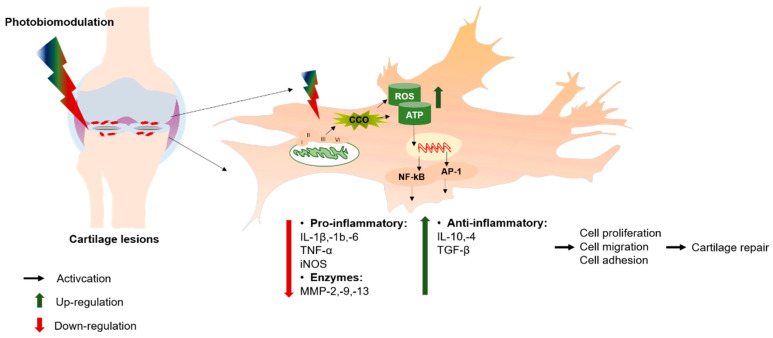
Schematic representation of photobiomodulation pathways in cartilage repair. PBM releases cytochrome-c oxidase (CCO), a key photoreceptor located in the mitochondrial respiratory chain at complex IV. The released CCO stimulates the mitochondrial respiratory chain, producing ROS and ATP through oxidative phosphorylation. This activates the NF-kB and AP-1 functions, leading to upregulation of proinflammatory cytokines (IL1β, -1b, -6), matrix metalloproteinase (MMP-2, -9, -13), and downregulation of anti-inflammatory cytokines (IL-10, -4, TGF-β), promoting cartilage repair.

The PBM-induced healing process was introduced by Endre Mester and National Aeronautics and Space Administration (NASA) researchers in the 1960s, which was proved to be capable of stimulating healing processes via the regulation of releasing proinflammatory and inflammatory cytokines (IL-1, -6, -1β, TNF-α, and iNOS), simultaneously, stimulating cell proliferation, migration, and differentiation via triggering adenosine triphosphate (ATP) and reactive oxygen species (ROS) production [19]. Nonetheless, the underlying mechanism for the abovementioned effectiveness remains unclear. One of the most well-known proposed mechanisms of PBM is in regard to the release of cytochrome-c oxidase (CCO) in the mitochondrion. The released CCO induces the functional changes in the original mitochondrial respiratory chain to generate ROS and ATP production through oxidative phosphorylation, which activates the NF-kB and AP-1 function to stimulate cell proliferation, migration, and adhesion (Figure 1) [20].

The CCO is located at complex IV of the mitochondrial respiratory chain and plays a critical role in cellular respiration, transferring four protons from CCO to one molecule of molecular oxygen to synthesize two water molecules, followed by producing ATP by translocating four protons across the mitochondrial membrane [21,22]. Photons dissociate inhibitory nitric oxide (NO), which is activated by the release of CCO in the mitochondria, resulting in increasing ATP production. Increased ATP synthesis may enhance wound healing since the release of oxygen from the body plays an essential role in wound healing processes, including oxidative killing bacteria, re-epithelialization, and angiogenesis [23].

During PBM, oxygen is transformed into reactive oxygen species (ROS), activating mitochondrial electron transport chains [24,25]. Mitochondrial reactive oxygen species (mROS) produce respiration products while simultaneously activating cellular signaling pathways. The functions of ROS as a low-level secondary messenger, transferring signals from extracellular signaling molecules and their membrane receptors to intracellular regulatory systems, result in regulating gene expression [26]. The cellular transcriptional response to ROS is primarily mediated by activating transcription factors NF-kB and AP-1, wherein NF-kB is mainly responsible for regulating immune and inflammatory responses [27]. When NF-kB is activated, it typically activates pro-inflammatory cytokines (IL-1, -2, -6, -8, -12, and TNF-α), pro-proliferative proteins (cyclin D1 and MYC), adhesion molecules (matrix metalloproteinases (MMP-2, -9, -13), inducible nitric oxide synthase (iNOS), angiogenic factors (vascular endothelial growth factor (VEGF)), and anti-apoptotic factors (Caspase-3 and B-cell lymphoma 2 (BCL-2)) [28]. Apparently, the activation of NF-kB regulates inflammatory responses by increasing inflammatory cytokines and adhesion molecules, and simultaneously regulating cell proliferation, migration, and differentiation, potentially healing cartilage damage [28].

### 2.2. In Vitro Studies of PBM Effects in Cartilage Regeneration

Research on PBM-induced cartilage regeneration at the cellular level is summarized in Table 1. Eleven in vitro studies were reviewed [9,10,12,13,29,30,31,32,33,34,35] with 10/11 studies using MSC-derived cells that were harvested from bone marrow, adipose tissue, and the umbilical cord of humans or rats. Herein, several light sources coupled with a range of wavelengths (415–910 nm) were applied for examining cell viability, proliferation, and differentiation, such as gallium-aluminum arsenide (GaAIAs), gallium arsenide (GaAs), light-emitting diode (LED), near infrared (NIR), and helium-neon (He-Ne). The effectiveness of PBM in regulating the expression of chondrogenesis genes and proteins and reducing inflammatory cytokines is summarized in Table 1.

In particular, fluences with a broad range from 0.75 to 180 J/cm^2^ were used with an independent combination of light sources and wavelengths, resulting in regulating cellular responses differently. According to de Abdrade et al., 2018, the use of PBM (InGaAIP, 660 nm, 180 J/cm^2^) inhibited the mitochondrial functions of human adipose mesenchymal stem cells (hADSCs), whereas 20 and 70 J/cm^2^ stimulated significant hADSC proliferation and increased mitochondrial activity in hADSC after 3 days of PBM treatment [9]. Similarly, Kan Yin et al., 2017 indicated that hADSCs showed enhanced proliferation after receiving a red laser of 660 ± 20 nm with 11 J/cm^2^ with a slight decrease observed at 16 J/cm^2^ [31]. In contrast, irradiation with low fluences (0.75 and 1.5 J/cm^2^, 660 nm, GaAIAs) had no impact on triggering VEGF and VEGFR-2 expression on hMSCs and rMSCs under low nutrient supplementation (5% FBS) [29]. Furthermore, the use of LEDs (3 J/cm^2^, blue, 415 nm, and green, 540 nm) demonstrated the downregulation of ATP level and inhibition of hADSC proliferation [12], Similarly, PBM (40 J/cm^2^, blue, 475 nm, and green, 516 nm) reduced GAG/DNA content and collagen type II compared to PBM (40 J/cm^2^, red, 635 nm) [34]. Meanwhile, PBM (GaAs, 910 nm, 8 J/cm^2^) reduced levels of inflammatory cytokines (IL-1β and IL-6) and DNA-binding activity of NF-κB in IL-1β-treated chondrocytes [35]. Similarly, a NIR laser (810 nm, 0.6–3 J/cm^2^) also showed an improved ATP production as well as cell viability, and proliferation after PBM [10,12].

In general, the most common wavelength in these in vitro studies is 600–660 nm, cited in 10/11 studies, which is combined with a range of fluences (3–12 J/cm^2^) capable of regulating ATP/ROS production, promoting cell viability, proliferation, and differentiation, and also reducing inflammatory cytokines. Moreover, PBM with a red or NIR (600–910 nm) laser provided greater efficacy in promoting cellular responses compared to LED (blue/green, 415–540 nm). Another significant finding is the optimal fluence range (3–10 J/cm^2^) for red or NIR lasers in promoting biological effects at the cellular level.

### 2.3. In Vivo Studies of PBM Effects in Cartilage Regeneration

Twenty animal studies were reviewed to evaluate the role of PBM in improving cartilage tissue healing, as summarized in Table 2 [36,37,38,39,40,41,42,43,44,45,46,47,48,49,50,51,52,53,54,55]. Among the studies, 17 used rats, 2 used mice, and 1 used rabbits to establish disease models. In particular, 17 studies with rat models included 7 of temporomandibular joint (TMJ) arthritis, 6 of osteoarthritis/knee osteoarthritis (OA/KOA), 2 of the monoiodoacetate (MIA) model of OA in rats (intra-articular injection of MIA), 1 of TMJ + OA, and 1 of KOA with ACL transection. The mouse models included two kinds of collagenase-induced arthritis (CIA) in RA and zymosan-induced arthritis. Additionally, the rabbit model involved the induction of folded ears. The table details disease models, laser parameters (e.g., light source, wavelength, power output/power intensity, irradiation time, and fluences), major outcomes, and references. Light sources varied, with 12 studies using GaAIAs lasers (808–940 nm, 3–57.14 J/cm^2^); 2 using LED (630 nm, 9–12 J/cm^2^), and others using YAG, InGaAs, and InGaAsP. Overall, these studies aimed to investigate PBM’s effects on joint arthritis with variations in light sources, wavelengths, and fluences.

As mentioned, GaAIAs lasers, using various wavelengths and fluences, were frequently employed in TMJ arthritis and KOA models in rats. As described in Table 2, wavelength and fluence have an adverse relationship in the same light source; particularly, the higher the wavelength, the lower the fluence. Studies of using PBM (GaAIAs, 808–810 nm, ~50 J/cm^2^) in TMJ or KOA in rats demonstrated positive results in reducing statistically significant reductions in inflammation after receiving PBM compared to control groups. According to Assis et al., 2016, reductions of IL-1b and MMP-13 were observed in groups undergoing irradiation (GaAIAs, 808 nm, 50 J/cm^2^), aerobic exercise, and both, whereas Caspase-3 indicated a reduction in groups combining aerobic exercise and irradiation [39]. Additionally, Assis et al., 2018 demonstrated that PBM, with or without aerobic exercise, significantly increased IL-10 and COL-II expression, as well as TGF-β levels [41]. Similarly, a decrease of IL-1b and caspase-3 was observed in groups receiving aquatic exercise coupled with or without PBM (GaAIAs, 808 nm, 50 J/cm^2^), whereas MMP-3 was only increasingly expressed after doing aquatic exercise and PBM [40]. Furthermore, PBM (GaAIAs, 830 nm) with a lower fluence of 3 J/cm^2^ resulted in reduced cartilage thickness and increased collagen fiber density in the articular region in TMJ arthritis compared to non-PBM and simultaneously showed a decrease in MMP-2 and MMP-9 activities [38]. Conversely PBM (GaAIAs, 940 nm, 500 mW) showed no significant improvement of cartilage, osteochondral junction, chondrocyte appearance, or subchondral ossification in the groups with and without PBM treatment [43], while PBM (InGaAsP, 940 nm, 20 mW) indicated moderate increase in GAG synthesis and reduction of caspase-3 in the PBM-treated group [42].

**Table 1:** Research on PBM effects for cartilage regeneration in vitro.

Objective	Laser Parameters					Outcome	References
	Light Sources	Wavelength	Power Output/Power Intensity	Irradiation Time	Fluency (J/cm^2^)		
**rMSCs and hMSCs**	GaAlAs	660 nm	30 mW	25 s 50 s 100 s 300 s	0.75 J/cm^2^ 1.5 J/cm^2^ 3 J/cm^2^ 9 J/cm^2^	PBM (3 J/cm^2^) triggered statistically significant VEGF and VEGFR2 expression on hMSCs and rMSCs under low nutrient supplement (5% FBS) compared other groups.	de Oliveria., 2014 [29]
**hUCMSCs**	Laser	635 nm 808 nm 1443 nm	200 mW	Twice/day for 3 days	12 J/cm^2^	PBM (635 nm) showed a greater impact on proliferation rate and ROS production compared to PBM of 808 and 635 + 808 nm. PBM (808 nm) stimulated the expression of IL-1, IL-6, and NF-kB, which was not in VEGF.	Chen et al., 2016 [30]
**hADSCs**	Red laser	660 nm ± 20	3-4.5 Mw	1-3 h	11-16 J/cm^2^	PBM (11 J/cm^2^) had a greater impact on cell proliferation compared to other fluences.	Kan Yin et al., 2017 [31]
**hADSCs**	InGaAIP	660 nm	40 mW	14 s 46 s 126 s	20 J/cm^2^ 70 J/cm^2^ 180 J/cm^2^	PBM (20 and 70 J/cm^2^) stimulated significantly cell proliferation after 3 days. Increase of mitochondrial activity was observed after 72 h. receiving PBM (20 and 70 J/cm^2^), where 180 J/cm^2^ inhibited mitochondrial functions.	de Abdrade et al., 2018 [9]
**hBMMSCs and hADSCs**	He-Ne red NIR	632.8 nm 630 nm 810 nm	0.5 mW 5 mW 5 mW	3 times at day 1, 3 and 5	0.6, 1.2, 2.4 J/cm^2^	Increase of cell viability (hADSCs/hBMMSCs) and decreased population doubling time (PDT) of hBMMSCs exhibited after irradiating at 630 nm. Combination of 630 and 810 nm promoted cell viability, decreased DPT and apoptosis of both cells.	Zare et al., 2019 [10]
**hADSCs**	Blue, green, red, NIR	415 nm 540 nm 660 nm 810 nm	NA	188 s	3 J/cm^2^	PBM (3 J/cm^2^, red/NIR) promoted ATP production and proliferation rate, while using blue/green wavelength downregulated ATP and inhibited cell proliferation.	Wang et al., 2017 [12]
**rADSCs**	GaAlAs	660 nm	30 mW	9 s 16 s 25 s	10 J/cm^2^ 18 J/cm^2^ 27 J/cm^2^	PBM (10 J/cm^2^) reduced DOX-induced toxicity effects on cell viability, apoptosis, and inhibited oxidative stress in rADSCs.	Lima et al., 2019 [13]
**Primary Chondrocyte in Rat**	HN-8318 He-Ne	632.8 nm	12 mW	8 min, daily treatment	5.74 J/cm^2^	PBM-treated chondrocytes significantly increased after 1 and 3 days, where GAGs level positively rose. Stimulation of PBM and IL-1β reduced TNF-α expression PBM upregulated protein (COL-II) and gene expression (ACAN, COLII, SOX-9).	Yang et al., 2020 [32]
**rBMMSCs**	He-Ne laser (helium-neon)	632.8 nm	1.7 mWt	15 min with 40-min pause	NA	An increase in chondrogenesis gene expression (TGF-β3, COL2A1, and SOX-9) was shown in two groups: laser and recombinant protein tgfβ3-based treatment. The laser-treated group exhibited chondrogenic differentiation capacity that was less significant than tgfβ3-based treatment.	Bozhokin et al., 2021 [33]
**hADSCs**	LED (blue, green, red)	475 nm 516 nm 635 nm	NA	10 min	40 J/cm^2^	PBM (red, 635 nm) triggered the increase in the size of pellet (3D) and gene expression (COL-2A1, IL-1β), while PBM (green and blue) reduced the pellet size (3D), GAG/DNA content, and collagen type II.	Schneider et al., 2021 [34]
**Normal human articular chondrocyte (NHAC-Kn)**	GaAs	910 nm	300 mW	256 s	8 J/cm^2^	PBM reduced inflammatory cytokine (IL-1β and IL-6) and DNA-binding activity of NF-κB in IL-1β-treated chondrocytes, where no impact on NF-kB responses in PBM-treated chondrocytes	Sakata et al., 2022 [35]

Abbreviation: PBM, photobiomodulation; hMSCs/rMSCs, human/rat mesenchymal stem cells; hADSCs/rADSCs, human/rat adipose stem cells; hBMMSCs/rBMMSCs, human/rat bone marrow mesenchymal stem cells; GaAIAs, gallium-aluminum-arsenide; GaAs, gallium arsenide; LED, light-emitting diode; NIR, near infrared; He-Ne, helium-neon; VEGF, vascular endothelial growth factor; VEGFR2, vascular endothelial growth factor receptor-2; FBS, fetal bovine serum; ROS, reactive oxygen species; IL-1, -6, and 1β, interleukin-1, -6, and 1β; NF-kB, nuclear factor kappa light-chain enhancer of activated B cells; TNF-α, tumor necrosis factor alpha; DOX, doxorubicin, ATP, adenosine triphosphate; GAGs, glycosaminoglycans.

Conversely, two studies evaluated the efficacy of laser devices (diode laser and laser moxibustion) and a couple more assessed LEDs in treating KOA, CIA, TMJ arthritis, and the MIA model of OA. The LED treatment (630 nm, 9 J/cm^2^) showed a significant increase in cartilage thickness relative to the OA group, but not the control group. Additionally, enzymatic activity of superoxide dismutase (SOD) and catalase (CAT) were higher in the LED-treated group than in the control group, while thiobarbituric acid-reactive substances (TBARS) were lower in both the control and OA groups [49]. In a similar investigation, Ryu et al., 2023 induced CIA in mice and then used LED (610 nm, 12 J/cm^2^) to evaluate inflammatory responses in fibroblast-like synoviocytes (FLSs) [55]. This study observed decreased TNF-α expression, and reduced proliferation, migration, and invasion in RA-FLSs, with downregulation of NF-kB and NLRP3 inflammasomes following PBM treatment [55]. Furthermore, the integration of PBM and methotrexate, an anti-rheumatic drug, significantly inhibited CIA progression [55]. Regarding laser devices, Peimani et al., 2014 used a diode laser device (Azor-2k, Russia) to promote healing of TMJ OA in rats, demonstrating improved cartilage repair but no effect on angiogenesis after 7 days of PBM [37]. Similarly, a laser moxibustion device (1500 J/cm^2^) applied for 10 min daily over 7 sessions reduced MMP-3, TNF-α, IL-1β, IL-6 levels, and the Osteoarthritis Research Society International (OARSI) score in MIA models compared to other treatment groups [46].

In summary, despite variations in laser parameters, PBM consistently showed positive effects on joint function, either alone or in combination with other treatments (e.g., drugs or exercise). These benefits were achieved through the regulation of immunoexpression (MMP-2, -3, -9, -13), inflammatory cytokines (IL-4, -10, -1β), and gene/protein expressions, ultimately enhancing the healing of TMJ arthritis and KOA in animal models.

### 2.4. Clinical Studies of PBM Effects in Cartilage Regeneration

In clinical studies, numerous assessment tools were used to evaluate patients with KOA/RA patients. Detailed, noninvasive investigations of arthritis symptoms were collected from patients via questionnaires and pain level examinations. These questionnaires assess osteoarthritis using the visual analogue scale (VAS), dolorimetry, numerical rating pain scale (NRPS), and osteoarthritis index (WOMAC) of Western Ontario and McMaster Universities [4]. Specifically, WOMAC provides information on symptoms and physical disabilities. VAS, dolorimetry, and NRPS have been widely used for assessing pain sensitivity, which ranges from “0” (no pain) to “10” (unbearable pain) [4]. For the assessment of deep muscular tissue sensitivity and muscle strength or balance, an assessment of pain pressure threshold (PPT) and functional reach test (FRT) were used, respectively. Moreover, evaluation of the motion and fundamental functions of the damaged joints in OA/RA patients is carried out by measuring the range of motion (ROM) or knee flexion range of motion (FROM). Additionally, it is necessary to investigate the changes in inflammatory factors (C-reactive protein (CRP) and interleukin-6 (IL-6), malondialdehyde (MDA) oxidative markers, and ATP production from the peripheral blood serum of patients, providing further evidence for repairing joint arthritis.

Eight clinical trials of assessing the therapeutic efficacy of PBM in KOA and RA patients over the past eight years (2016–2023) are summarized in Table 3, including six cases of KOA and two cases of RA [56,57,58,59,60,61,62,63]. Among these clinical trials, five utilized a wavelength of 1064 nm, known as the longest wavelength in high-intensity (>0.5 W) laser therapy (HILT) derived from two difference light sources. Four applied a pulsed Nd: YAG laser and one applied a semiconductive neodymium laser IV [64]; one used GaAIAs (860 nm, 7.5 J/cm^2^); one used laser acupoints (808 nm, 7.5 J/cm^2^); and one used a super-pulsed laser (950 nm), IR LEDs (875 nm), and red LEDs (640 nm). In most cases, KOA/RA patients received HPL-PBMT with multiple sessions and different fluences for each session.

Notably, two laser sources (semiconductive neodymium laser IV and pulsed Nd: YAG laser, ASA, Arcugnano, Vicenza, Italy) coupled with the same wavelength and fluence (12 and 120 J/cm^2^) in KOA patients exhibited an encouraging outcome [56,61]. A single application per day in the first three sessions of 12 J/cm^2^ for 2 min and further sessions of 120 J/cm^2^, 10 min targeting medial and lateral areas showed a significant reduction in pain as measured by VAS; and dolorimetry significantly improved after 7 days’ treatment that was maintained within 3 months. Simultaneously, the laser-treated group showed a decrease in contact surface area (pedobarometric analysis), resulting in a balance between two legs in static and dynamic positions [56]. Similarly, Akaltun et al., 2020 demonstrated that the integration of HILT and exercise therapy (ET) resulted in greater improvement in VAS, WOMAC scores, FCT, and FROM after 2 weeks of irradiation; however, VAS and WOMAC scores continued to decrease until the 6th week compared to the placebo laser (PL) + ET group, and a significant improvement in cartilage thickness was obtained after 6 weeks of receiving HILT plus ET [61]. Following the clinical protocol of Akaltun et al., 2020, Burak et al., 2023 reported a similar positive outcome in reducing pain levels and enhancing FROM, muscle strength, WOMAC, and femoral cartilage thickness (FCT) in both HILT/ET and sham laser/ET-based treatments [61,63]. Instead of using HILT, Afnan et al., 2018 applied a GaAIAs laser (860 nm, 7.5 J/cm^2^) to treat 40 RA patients; the obtained results indicated a statistical difference in elbow extension ROM (range of motion) between pre- and post-treatment in the laser-treated group, whereas there was no difference in elbow flexion ROM [60]. In 2022, Afnan and his team further confirmed the effects of laser acupoints (808 nm, 7.5 J/cm^2^) in RA patients. Specifically, the treated group (laser + methotrexate + ET) significantly enhanced CRP, IL-6, malondialdehyde (MDA) oxidative marker, and ATP compared to control group (methotrexate + ET) [62]. To better understand the dependent fluences, the study of Nazari et al., 2019 investigated the impact of HILT with a moderate fluence of 60 J/cm^2^ instead of 120 J/cm^2^ [58]. Results indicated an increase in knee FROM, improved WOMAC scores, and a reduction in VAS after both treatment and a 12-week follow-up.

**Table 2:** Research on PBM effects of cartilage regeneration in vivo.

Objective	Laser Parameters					Disease Model (In Vivo)	Outcome	References
	Light Sources	Wavelength (Nm)	Power Output/Power Intensity	Irradiation Time	Fluency (J/cm^2^)			
**Chondrocytes (Rat)**	GaAlAs	810 nm	20 mW	60 s/session 7 sessions within 2 weeks	51.02 J/cm^2^	TMJ in rats	Reduced inflammation after receiving PBM and steroid treatments statistically compared to control group.	Khozeimed et al., 2014 [36]
**Chondrocytes (Rat)**	Diode laser device (Azor-2k, Russia)	880 nm	100 mW	10 min/day within 7 days	NA	TMJ arthritis in rats	Improvement of damaged cartilage and non-impact in angiogenesis observed after 7 days’ PBM.	Peimani et al., 2014 [37]
**Chondrocytes (Rat)**	GaAlAs	830 nm	30 mW	12 s/session 7 sessions/48 h within 13 days	3 J/cm^2^	TMJ arthritis in rats	The PBM-treated group exhibited reduced thickness and higher collagen fibers in the articular region compared to non-PBM and simultaneously showed a decrease in MMP-2 and MMP-9 activities.	Lemos et al., 2016 [38]
**Chondrocytes (Rat)**	GaAIAs	808 nm	50 mW	28 s, 3 days/ weeks for 8 weeks	50 J/cm^2^	KOA in rats	Reduction of IL-1b and MMP-13 shown in groups undergoing irradiation, aerobic exercise, and both, whereas caspase-3 indicated a reduction in groups combining aerobic exercise and irradiation.	Assis et al., 2016 [39]
**Chondrocytes (Rat)**	GaAIAs	808 nm	50 mW	28 s, 3 days/ weeks for 8 weeks	50 J/cm^2^	KOA in rats	A decrease of IL-1b and caspase-3 was exhibited in groups receiving aquatic exercise coupled with/without PBM, whereas MMP-3 was only increasingly expressed after aquatic exercise and PBM.	Milares et al., 2016 [40]
**Chondrocytes (Rat)**	GaAIAs	808 nm	50 mW	28 s, 3 days/ weeks for 8 weeks	50 J/cm^2^	KOA in rats	PBM/non-PBM coupled with aerobic and exercise training performed increasingly in IL-10 and COL-II expression, where TGF-β levels were promoted with and without exposing PBM combined with aerobic exercise.	Assis et al., 2018 [41]
**Chondrocytes (Rat)**	Gallium arsenidephosphide (InGaAsP)	940 nm	20 mW	60 s/session, 7 sessions, within 2 weeks	NA	OA in TMJ of rats	The moderate increase of GAGs and reduction of caspase-3 were illustrated in the PBM-treated group.	AbuBakr et al., 2018 [42]
**Chondrocytes (Rat)**	GaAIAs	940 nm	500 mW	14 sessions/48 h, then 28 sessions/24 h	15 J/cm^2^	TMJ arthritis in Rat	No significant difference in improvement of cartilage, osteochondral junction, chondrocyte appearance, or subchondral ossification in the groups with and without PBM treatment.	Memis et al., 2018 [43]
**Chondrocytes (Rat)**	GaAIAs	808 nm	50 mW	28 s, 3 days/ weeks	50 J/cm^2^	KOA in rats	Improvement in chondrocyte density of the treated-groups (PBM, chondroitin and glucosamine sulfate (CS/GL), PBM/CS/GL) statistically compared to control group. Group of PBM/CS+GL upregulated significantly inflammatory cytokine of COL-II, and downregulated IL-1β, also exhibited a decrease in OARS score compared to control group.	Shanches et al., 2018 [44]
**Chondrocytes (Rat)**	GaAIAs	830 nm	30 mW	5–20 J/cm^2^ (20–80 s) 10 sessions/ 48 h, within 3 weeks	5, 10, 20 J/cm^2^	TMJ arthritis in rats	PBM (5 J/cm^2^) reduced significantly the thickness of articular cartilage at the middle region and increased the GAGs concentration compared other fluences. Both PBM-treated groups showed a greater reduction in MMP-9 and MMP-2 activities; and IL-1β concentration compared to the group of TMJ arthritis.	Lemos et al., 2020 [45]
**Chondrocytes (Rat)**	Laser Moxibustion Device	10.6 µm	NA	10 min/day, total of 7 times irradiation	1500 J/cm^2^	MIA model of OA in rat	Reduction of immunoexpression (MMP-3), protein expression (TNF-α, IL-1β, IL-6), and OARSI score detected in MIA/laser-based treatment compared to other treated-groups (MIA and MIA/sham laser).	Li et al., 2020 [46]
**Chondrocytes (Rat)**	GaAIAs	850 nm	200 mW	30 s	NA	KOA in rats	PBM stimulated the increase of COL-II and TGF-β expression in rat OA model. Abnormal chondrocyte organization was observed in the PBM-treated group after histological analysis.	Trevisan et al., 2020 [47]
**Chondrocytes (Rat)**	GaAIAs	850 nm	100 mW	40 s/site, 4 sites	57.14 J/cm^2^	MIA model of OA in rat	Non-PBM efficacy in movable activities, but regeneration of damaged cartilage was proved in superficial layers after PBM.	Balbinot et al., 2021 [48]
**Chondrocytes (Rat)**	LED	630 nm	300 mW	30 s 3 times/ week for 8 weeks	9 J/cm^2^	KOA in rats	Cartilage thickness of LED-treated group (LEDG) exhibited significantly higher values compared to that in the OA group (OAG), but no difference compared to the control group (CG). The enzymatic activity of superoxide dismutase (SOD) and catalase (CAT) of LEDG was higher than that of CG, and thiobarbituric acid-reactive substances (TBARS) lower than that of CG and OAG.	Lorena et al., 2021 [49]
**Chondrocytes (Rat)**	GaAIAs	808 nm	50 mW	16 s (in vitro) 28 s (in vivo), 3 days/ week for 4 and 8 weeks	28 J/cm^2^ (in vitro) 50 J/cm^2^ (in vivo)	KOA in rats (in vitro and in vivo)	PBM enhanced chondrocyte proliferation significantly after 3 days in vitro. An increase of IL-4, IL-10, and COL-II expression was proved after 8 weeks of PBM, whereas a decrease of inflammatory cytokine (IL1-β) was detected after PBM-treated cartilage in vivo. PBM stimulated an increase in TGF-β, COL-2, and aggrecan expression after 4 weeks, whereas that was not observed after 8 weeks in vivo.	Tim et al., 2022 [50]
**Chondrocytes (Mice)**	GaAlAs	830 nm	10 mW	15 s and 150 s 4 treatment sessions	3 and 30 J/cm^2^	Zymosan-induced arthritis in mice	A decrease in MMP-9 and an increase in TIMP-2 were indicated after exposing PBM. PBM of 3 J/cm^2^ expressed a level of MMP-2, and -9 significantly lower than that of 30 J/cm^2^; there was no significance in the MMP-13 level and a higher level in MMP-14.	Lucia et al., 2022 [51]
**Chondrocyte (Rabbit)**	YAG laser	1064 nm	NA	once/day on day 0, 2 and 4	80 J/cm^2^	Rabbit with the folded ears (in vitro and in vivo)	PBM upregulated aromatase (Cyp19) expression and increased collagen synthesis in chondrocytes in vivo. The estrogen rate in the cartilage tissue was significantly increased after PBM via the activation of Cyp19, which promoted chondrocyte proliferation and collagen synthesis.	Zhu et al., 2023 [52]
**Chondrocytes (Rat)**	NA	NA	NA	48 h for 7 days (4 sessions)	38 J/cm^2^	TMJ arthritis in rats	Articular disc thickness, condylar cartilage thickness, and TMJ structure were improved after three weeks’ PBM compared to the arthritic group, with improvement in cartilage tissue and less osteochondral detachment.	Rana et al., 2023 [53]
**rADSC/ ADC-derived secretome**	InGaAs	980 nm	500 mW	60 s	38 J/cm^2^	TMJ arthritis in rats	Groups of ADSCs or secretome-based treatments exhibited an improvement in joint structure, articular disc, and condylar cartilage thickness; simultaneously, a reduction of TNF-α was detected after three weeks’ PBM compared to the arthritic group. No significance between using ADSCs and ADSC-derived secretome for improving TMJ arthritis in rats.	Rana et al., 2023 [54]
**Fibroblast-like synoviocytes from RA patients (RA-FLSs)**	LED	610 nm	5 and 10 mW/cm^2^	20 min	12 J/cm^2^	Collagen-induced arthritis in mouse (CIA)	TNF-α expression was decreased in proliferation, migration, and invasion in RA-FLSs, whereas NF-kB and NLRP3 inflammasomes were downregulated after receiving PBM. Integration of PBM and methotrexate, an anti-rheumatic drug, contributed to inhibiting CIA development.	Ryu et al., 2023 [55]

Abbreviation: PBM, photobiomodulation; OA/KOA, osteoarthritis/knee osteoarthritis; TMJ, temporomandibular joint; CIA, collagen-induced arthritis; MIA, monoiodoacetate; ADSCs, adipose stem cells; GaAIAs, gallium-aluminum-arsenide; LED, light-emitting diode; IL-1b, -6, -10, and 1β, interleukin-1b, -6, -10, and 1β; NF-kB, nuclear factor kappa light chain-enhancer of activated B cells; GAGs, glycosaminoglycans; TNF-α, tumor necrosis factor-alpha; TGF-β, transforming growth factor beta; MMP-2, -9, -13, and -14, matrix metalloproteinase-2, -9, -13, and -14; OARS, oxford arthroplasty early recovery score; OARSI, Osteoarthritis Research Society International; TIMP-2, tissue inhibitor of metalloproteinases 2; Cyp19, human aromatase p450; NLRP3, NOD-, LRR-, and pyrin domain-containing protein 3.

Nevertheless, there were no differences in the Timed Up and Go test (TUG), 6 min walk test (6MWT), or WOMAC pain subscale between HILT and conventional (TENS/US) treatment, but HILT showed greater improvement compared to exercise therapy alone (ET) [58]. Additionally, Gomes et al., 2018 explored the PBM effects under various laser sources, including super-pulsed lasers (905 nm), IR LEDs (875 nm), and red LEDs (640 nm), in 60 KOA patients [59]. The data revealed that the group receiving PBM combined with exercise showed reduced NRPS scores compared to other groups (exercise alone and exercise with placebo PBM), while other metrics, such as PPT and FRT tests, did not show clinically significant differences.

In summary, these clinical trials mainly focused on using HILT (1064 nm and pulsed Nd: YAG laser) in conjunction with a wide range of fluences (12–120 J/cm^2^), which apparently can reduce joint arthritis in OA and RA patients. Nonetheless, due to the high degree of similarity among PBM protocols in existing studies, the broader therapeutic effects of PBM (particularly on musculoskeletal function and tissue remodeling) remain inadequately characterized. As such, further investigations exploring a wider range of fluence parameters, laser modalities, and wavelengths are necessary to fully delineate the clinical utility of PBM in arthritis management.

In parallel with the need for protocol diversification, methodological advancements are also warranted in outcome assessment. While most PBM clinical studies focus on symptom-based and biochemical endpoints, the integration of structural imaging remains limited. Magnetic resonance imaging (MRI), in particular, offers a noninvasive and objective means of evaluating cartilage morphology, matrix integrity, and joint structure over time. Despite its diagnostic value, MRI is seldom incorporated into PBM research. Future clinical trials should aim to standardize the use of advanced MRI protocols, such as T2 mapping and delayed gadolinium-enhanced magnetic resonance imaging of cartilage (dGEMRIC) to rigorously assess the regenerative potential of PBM and enhance its translational relevance through high-resolution, quantifiable structural data.

### 2.5. PBM Strategies on Cartilage Regeneration-Related Studies

Since PBM promotes cartilage regeneration through various approaches such as in vitro, in vivo, and clinical trials, it is crucial to establish specific protocols tailored to each approach. A total of 39 studies including in vitro, in vivo, and clinical trials were selected for this review, which provided valuable summarized information for both researchers and doctors to narrow down the range of variables investigated, resulting in determining procedures compatible with their specific experimental conditions.

**Figure 2:**
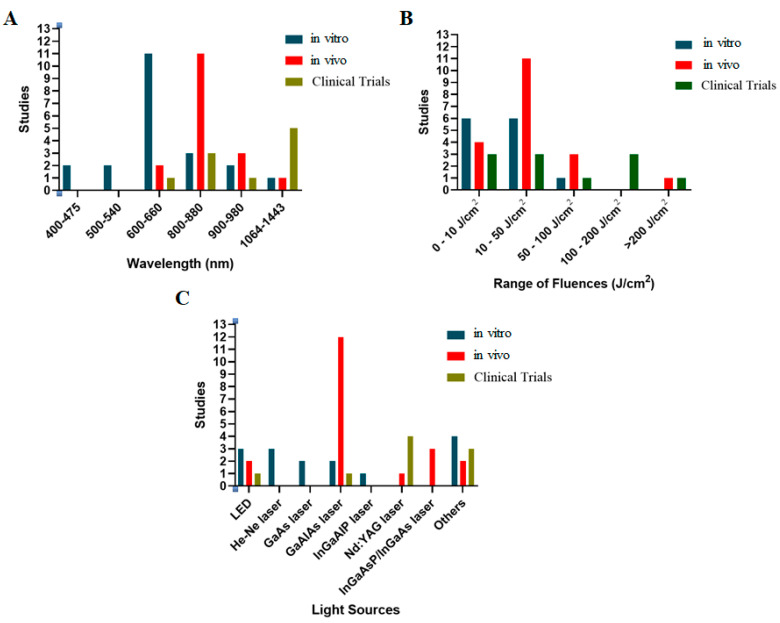
Dependent wavelengths (**A**), fluences (**B**), and light sources (**C**) of PBM protocols for cartilage regeneration in vitro, in vivo, and in clinical trials. Generally, GaAIAs lasers (800–880 nm, 10–50 J/cm^2^), and Nd: YAG lasers (1064 nm, 0–50 and 100–200 J/cm^2^) were predominantly used in vivo and clinical approaches, respectively. Additionally, various light sources were utilized in cellular investigation.

In terms of in vitro studies (shown in Table 1), 12 studies were collected, and 4 of these employed multiple light sources and wavelengths for comparative analysis. Notably, a range of wavelengths was broadly applied for cartilage-related studies from 600 to 660 nm with different light sources, such as He-Ne, GaAIAs, red laser, and red LED (Figure 2A,C). Regarding the fluences of PBM, a common fluence range used was from 0 to 50 J/cm^2^.

Moreover, while animal experiments tend to use GaAIAs lasers associated with a range of wavelengths of 800–880 nm, clinical studies predominantly used the 1064 nm wavelength from the pulsed Nd: YAG laser, which offers deeper tissue penetration compared to other laser types. Herein, the fluences used for KOA/RA patients were significantly higher, in the range 100–200 J/cm^2^, while cellular and animal studies used 0–50 J/cm^2^ and 10–50 J/cm^2^, respectively (Figure 2).

Apparently, protocols for PBM were different among studies, which could be attributed to the size, location, and depth of the affected regions. Additionally, the longer the wavelength, the deeper penetration into the targeted tissue. Besides, the choice of an appropriate light source is critical for accelerating and enhancing the healing process. For instance, Schneider et al., 2021 indicated PBM (40 J/cm^2^, blue, 475 nm, and green, 516 nm) reduced GAG/DNA content and collagen type II compared to PBM (40 J/cm^2^, red, 635 nm) [34]. Thus, different light sources elicited varied cellular responses. On the other hand, additional influencing can significantly modulate biological effects and tissue repair process. For example, de Andrade et al., 2019 reported increased mitochondrial activity following PBM exposure at 20 and 70 J/cm^2^, while a higher fluence 180 J/cm^2^ inhibited mitochondrial functions [9].

By examining the effects of PBM on cartilage-related cells and conditions across these 39 studies, from cellular models to clinical trials, researchers and clinicians can more effectively refine treatment parameters. Despite the encouraging outcomes observed, the precise biological mechanisms underlying PBM remain incompletely understood and warrant further investigation.

**Table 3:** Research on PBM effects of cartilage regeneration in clinical trials.

Objective	Laser Parameters					Disease Model (In Vivo)	Outcome	References
	Light Sources	Wavelength (nm)	Power Output/Power Intensity	Irradiation Time	Fluency (J/cm^2^)			
**Human**	Semiconductive neodymium laser IV	1064 nm	12,000 mW	Single application/day, first three sessions, 2 min (medial and lateral area) Next 4 sessions, 10 min (medial area)	12 J/cm^2^ 120 J/cm^2^	72 patients (Pain in KOA)	Reduction in pain level (visual analogue scale (VAS) and dolorimetry) significantly improved in the laser-based treatment after 7 days and was maintained over 3 months. Laser-treated group showed a decrease in contact surface area (pedobarometric analysis), resulting in a balance between two legs in static and dynamic positions.	Angelova et al., 2016 [56]
**Human**	Pulsed Nd:YAG laser	1064 nm	10,500 mW	Twice/ week for 6 weeks	5100–17,800 J/cm^2^	67 patients (KOA)	Pain levels were measured by VAS and Western Ontario and McMaster Universities Osteoarthritis (WOMAC) questionnaire, which were significantly reduced in all groups after 6 weeks. The treated group of HILT (laser) + glucosamine/chondroitin sulfate + exercise downregulated synovial thickness (ST), while there was no significance in femoral cartilage thickness (FCT).	Alayat et al., 2017 [57]
**Human**	Pulsed Nd:YAG laser	1064 nm	5000 mW	8 min, 12 sessions 3 times/ week for 12 weeks	60 J/cm^2^	93 patients (KOA)	HILT (laser) and exercise-based treatment increased knee flexion range of motion (FROM), improved WOMAC scores, and reduced the VAS after both treatment and 12 weeks. There was no difference in Timed Up and Go test (TUG), 6 min walk test (6MWT), or WOMAC pain subscale between HILT and conventional (TENS/US) treatment, but greater improvement compared to alone exercise therapy (ET).	Nazari et al., 2019 [58]
**Human**	1 superpulsed laser 4 IR LEDs 4 red LEDs	905 nm 875 nm 640 nm	2.25 mW/cm^2^ 77.76 mW/cm^2^ 66.64 mW/cm^2^	Twice a week/5 weeks, plus exercise	0.12 J/cm^2^ 4.46 J/cm^2^ 4 J/cm^2^	60 patients (KOA)	The treated group of PBM plus exercise reduced the numerical rating pain scale (NRPS) compared to other groups (exercise and exercise/placebo PBM), whereas other responses including pressure pain threshold (PPT) and muscle strength or balance (functional reach test (FRT)) had a non-clinical significant difference.	Gomes et al., 2018 [59]
**Human**	GaAlAs	860 nm	100 mW	3 sessions/week for 4 weeks	7.5 J/cm^2^	40 patients (RA)	In the laser-treated group, a statistical difference in elbow extension ROM (range of motion) between pre- and post-treatment was detected, whereas there was no difference in elbow flexion ROM.	Afnan et al., 2018 [60]
**Human**	Pulsed Nd:YAG laser	1064 nm	12,000 mW	5 sessions/ week, for 2 weeks 3 first sessions with 2 min 7 following sessions, 10 min	12 J/cm^2^ 120 J/cm^2^	54 patients (KOA)	HILT + ET integration indicated an greater improvement in VAS, WOMAC scores, FCT, and FROM after 2 weeks of irradiation; however, VAS and WOMAC scores were detected decreasingly in the 6th week compared to placebo laser (PL) + ET group. A significant improvement in cartilage thickness was obtained after 6 weeks receiving HILT plus ET.	Akaltun et al., 2020 [61]
**Human**	Laser acupoints	808 nm	100 mW/cm^2^	60 s	7.5 J/cm^2^	60 patients (RA)	Treated group (laser+methotrexate+ET) showed significantly enhanced C-reactive protein (CRP), IL-6, malondialdehyde (MDA) oxidative marker, ATP compared to control group (methotrexate+ET).	Afnan et al., 2022 [62]
**Human**	Pulsed Nd:YAG laser	1064 nm	10,000 mW 5000 mW	3 sessions/first week, 2 min 6 sessions within following 2 weeks, 10 min	12 J/cm^2^ 120 J/cm^2^	60 patients (KOA)	An improvement in reducing pain level and increasing FROM, muscle strength, WOMAC, and FCT in both HILT/ET-, and sham laser/ET-based treatment.	Burak et al., 2023 [63]

Abbreviations: PBM, photobiomodulation; KOA, knee osteoarthritis; RA, rheumatoid arthritis; GaAIAs, gallium-aluminum-arsenide; LED, light-emitting diode; VAS, visual analogue scale; HILT, high-intensity laser therapy; ET, exercise therapy; GCS, glucosamine/chondroitin sulfate; TENS/US, transcutaneous electric nerve stimulation/ultrasound; FROM, flexion range of motion; ROM, range of motion; FCT, femoral cartilage thickness; ST, synovial thickness; NRPS, numerical rating pain scale; WOMAC, Western Ontario and McMaster Universities Osteoarthritis.

## 3. PBM in Cartilage Tissue Engineering

### 3.1. Cartilage Tissue Engineering

To repair articular cartilage, there are three common surgical strategies, including bone marrow stimulation (microfracture or subchondral drilling), allografts and autografts, and cell therapies. In this review, we focused on cell-based therapies for cartilage regeneration. These cells can be implanted into the lesion or administered via intra-articular injection depending on the type of AC defects. Nevertheless, intra-articular injection has been shown to carry higher risks compared to autologous cell implantation (ACI) in terms of cell migration to the non-target tissues. To enhance cell-based therapies, tissue engineering approaches have been developed to retain the cells at the implantable site, in which cells are encapsulated in the scaffold. Though cartilage tissue engineering (CTE) has proven to be a promising strategy for cartilage repair and regeneration in numerous previous studies, CTE presents specific challenges. For one, due to the unique and complex structure of cartilage, the selection of a proper biomaterial is highly critical to construct an engineered tissue that integrates seamlessly with the AC defects. Furthermore, selecting an appropriate cell type for encapsulation within engineered tissue significantly enhances cartilage repair.

#### 3.1.1. Cell Sources

Selection of a proper cell line in cartilage tissue engineering (CTE) plays an important role in finding a potent regenerative cell source, although it remains underexplored [65]. In recent years, some cell sources, including differentiated cells (articular and nasal chondrocytes), and stem cells (bone marrow mesenchymal stem cells (BMMSCs) and adipose mesenchymal stem cells (ADSCs)) have been studied for promoting cartilage repair [65].

##### Chondrocytes

Chondrocytes are resident cells in cartilage, and depending on their origin, they have different functions. Since this review focused on OA and RA diseases, the roles and functions of AC-derived chondrocytes are highlighted. Specifically, AC-derived chondrocytes are in charge of synthesizing and regulating extracellular matrix (ECM) components, including collagen, proteoglycans (PG), glycoproteins, and hyaluronan [66]. The network of ECM plays a vital role in balancing compressive forces in AC, thereby modulating intracellular signal transduction and chondrocyte adhesion [67]. The two most common phenotypic markers specific to AC are collagen II and aggrecan [67]. Meanwhile, collagen II serves as the primary structural protein in the cartilage ECM, while aggrecan (ACAN) is a proteoglycan containing sulfated glycosaminoglycan (GAG) chains, including chondroitin sulfate (CS) and keratan sulfate (KS), and is responsible for supplying water and viscoelasticity to the musculoskeletal system [67]. The first surgical method employing chondrocytes was autologous chondrocyte implantation (ACI), which resulted in periosteum hypertrophy in approximately 22% of patients [68]. The second generation of this surgical approach is matrix-assisted chondrocyte implantation (MACI), which involves encapsulating the chondrocytes in a collagen-based scaffold before implantation [69]. Though both procedures improve AC abnormalities, patients have to endure two surgeries (harvesting chondrocytes and reimplanting them into defects), which creates a financial burden, and requires a longer recovery time [15]. Moreover, because chondrocytes frequently lose chondrogenic markers during culture expansion, their lifespan is impacted, resulting in decreasing treatment effectiveness [69].

##### Mesenchymal Stem Cells

Mesenchymal stem cells (MSCs) are widely recognized as an alternative candidate for promoting cartilage regeneration due to their self-regeneration and differentiation: they can differentiate into chondrocytes, osteocytes, adipocytes, and other lineages [70,71,72]. MSCs can synthesize ECMs and recruit endogenous MSCs to the defect site through the release of cytokines and growth factors, which is beneficial for enhancing the repair of AC lesions. Similarly, surgical strategies, such as autologous MSC implantation (AMSCI) and matrix-induced AMSCI, either through direct implantation or intra-articular injection, are viable strategies for treating damaged-AC [1]. Herein, matrix-assisted MSC implantation (MAMSCI) has demonstrated superior retention of implanted MSCs at the target region compared to intra-articular MSC injection, thereby enhancing the healing process of AC defects [73]. The two preferred candidates of MSCs for repairing damaged AC are bone marrow (BM-MSCs), and adipose tissue (ADSCs) [1]. The utilization of BM-MSCs is limited due to their low abundance and harvesting quantities, as well as the difficulties of isolating them via iliac crest aspiration. Additionally, the role of BM-MSCs is similar to that of osteogenic progenitors; hence, they prefer to undergo osteogenic differentiation [74]. Furthermore, ADSCs may be extracted from adipose tissues with less invasiveness and pain and they have similar regenerative properties to other MSCs, such as better progenitor differentiation potential, making them a favorable cell choice [74,75,76].

### 3.2. PBM-Induced Biological Effects Supporting Effective Cartilage Tissue Engineering

Several approaches have been discovered to maintain viable 3D cultures. Some focused on nutrient supplementation, while others utilize growth factors, hypoxia priming, and preconditioning to boost cell growth and survival [17]. PBM has been used as a light preconditioning agent in 3D culture system, promoting cell survival, proliferation, and differentiation [17]. Notably, cellular metabolism in the engineered-tissues is primarily influenced by their biomaterial characteristics, which include material, structure, and mechanics [17].

In this context, nine studies (from 2015 to 2024) were reviewed regarding the integration of PBM and CTE to assess the efficacy of PBM application to CTE in promoting cartilage regeneration, as summarized in Table 4. There were seven in vitro studies, with five of them investigating the potential chondrogenic differentiation of stem cells, and others investigating chondrocyte proliferation and migration in 3D scaffolds. The summarized data indicated that both CO_2_ laser (10,600 nm, 1.2–4.8 J) and the Er:YAG laser (2940 nm, 0.5 J) enhanced chondrocyte growth on decellularized extracellular matrix (DECM) scaffolds [77]. Nonetheless, the presence of thermal denaturation prevented chondrocytes from migrating in the CO_2_ laser-treated scaffold compared to Er:YAG laser-treated scaffolds [77].

Similarly, Hang et al., 2024 demonstrated that PBM using a near-infrared (NIR) laser (830 nm, 5 J/cm^2^) enhanced chondrocyte proliferation, glycosaminoglycan (GAG) synthesis, and extracellular vesicle (EV) release in alginate scaffolds [18]. Furthermore, multiple 3D culture platforms were utilized to investigate the potential chondrogenic differentiation of various stem cells, including human dental pulp stem cells (hDPSCs), rabbit mesenchymal stem cells (rMSCs), human adipose stem cells (hASCs), Wharton’s jelly mesenchymal stem cells (WJ-MSCs), and meniscus-derived stem cells (MeSCs). Zaccara et al., 2018 demonstrated PBM effects (660 nm, 3.3 J/cm^2^) of two groups (PBM/5% FBS and PBM/10% FBS) significantly accelerated chondrogenic differentiation in 3D agarose gel culture compared to the control group (10% FBS) at day 14 [78]. Similarly, GAG production in differentiated MeSCs increased considerably following PBM (660 nm, 4 J/cm^2^) and was significantly higher than in the control group [79].

In contrast, Muneekaew et al., 2022 found that incorporating chemical factors was less effective than using extrinsic PBM (EPM) and collagen microislands for inducing adipogenic differentiation [80]. In 2016, Fekrazad et al. investigated the PBM effect (810 nm, 4 J/cm^2^) and BMSCs-seeded type I collagen scaffold on cartilage repair in rabbits. The data exhibited no significant difference in new cartilage formation and inflammation among the groups, but new bone formation was significantly observed [81].

Further work by Fekrazad and colleagues (2019) confirmed that the chondrogenic response to PBM is wavelength-dependent. In general, the IR laser exhibited a greater stimulatory effect on chondrogenesis-related gene expression (Col 10) than the red/green/blue lasers. Notably, a combination of IR laser and blue laser could promote the expression of SOX-9, Col 2, and aggrecan [82].

In addition, PBM (808 nm, 50 mJ/cm^2^) combined with chondrocyte-seeded chitosan hydrogel inhibited articular degeneration by boosting TGF-β, decreasing TNF-α, and enhancing Col 2 expression in OA Wistar rats [83]. On the other hand, the Erbium: glass laser impregnated with starch-stabilized magnetite nanoparticles (SSNPs) contributed to decreasing acid proteoglycan content in costal and articular cartilage [84].

Collectively, these findings support PBM as a practical and effective strategy for enhancing cellular responses in scaffold-based 3D culture systems designed for cartilage tissue engineering.

## 4. Conclusions and Prospects

In conclusion, this comprehensive analysis clearly highlights PBM’s potential for stimulating molecular and cellular pathways that promote cartilage repair in AC abnormalities. The extensive review begins by emphasizing the potential of PBM-based therapies as regenerative medicine due to their drug-free, noninvasive, and safe nature, which can reduce inflammation in AC defects while simultaneously promoting cell proliferation, migration, and chondrogenic differentiation. Nonetheless, the underlying mechanism for the aforementioned efficacies remains to be fully understood due to the independence in employing diverse light sources (lasers or LEDs), wavelengths (green, blue, red, NIR), and fluences (low, medium, or high) on various PBM-treated targets. Hence, by analyzing the trends in PBM-based therapies to the specific targets in cartilage regeneration, this detailed review may assist scientists and physicians in optimizing their PBM-based therapy approaches. Furthermore, this review underlined the contribution of PBM in CTE, which appears to be a promising strategy for accelerating and optimizing cartilage regeneration. Consequently, further research should be performed to standardize PBM protocols, both with and without CTE, to maximize therapeutic outcomes.

**Table 4:** A combination of PBM and tissue engineering/biomaterial in cartilage regeneration.

Objective	Laser Parameters					Biomaterials/Tissue Engineering	Cell Sources/Animal Model	Main Outcome	References
	Light	Wavelength (Nm)	Power Output/Power Intensity	Time	Fluency				
To determine the Erbium:glass laser effect on costal and articular cartilage impregnated with SSNPs.	laser septochondrocorrection	1.56 µm	0.7 W	5 s	NA	SSNPs	Porcine articular and costal cartilage disks	SSNPs can be safely used for the laser diagnostics and therapy.A slight decrease in acid proteoglycan content occurs after laser treatment, but the collagen network remains unchanged.	Soshnikova et al., 2015 [84]
Evaluate an application of PBM and BMSCs-seeded type I collagen-scaffold on cartilage repair in the rabbits	Ga-Al-As	810	NA	Every other day for three weeks	4 J/cm^2^	Type I collagen scaffold	AC defect in rabbits	No significant difference in new cartilage formation and inflammation among the groups, but new bone formation was significantly found.	Fekrazad et al., 2016 [81]
Investigating the modification of dECM scaffold’s surface using CO_2_ and Er:YAG lasers to improve chondrocyte migration within scaffolds.	CO_2_ laser (AcuPulse) Er:YAG laser (SupErb XL)	10.600 nm 2.940 nm	8 W NA	0.03–0.06 s 0.1–1.0 ms	1.2–4.8 J 0.5 J	DECM scaffold	Chondrocyte	Both laser types result in increased scaffold surface area and cell growth.Chondrocytes were inhibited to migrate in the CO_2_ laser-treated scaffold compared to Er:YAG laser-treated scaffold due to the present of thermal denaturation.	Eva et al., 2018 [77]
Evaluate PBM impacts on the differentiated potential of hDPSCs in 3D agarose gel culture	InGaAlP diode laser	660	NA	10 s	3.3 J/cm^2^	3D Agarose gel culture	hDPSCs	PBM/5% FBS and PBM/10% FBS significantly accelerated chondrogenic differentiation in 3D agarose gel culture compared to the control group (10% FBS) at day 14, but there was no significant difference between the two PBM groups on days 7 and 14.	Zaccara et al., 2018 [78]
Evaluate wavelength dependence on promoting chondrogenic differentiation potential of rMSCs.	IR laser, red laser, green laser, blue laser	810 660 532 475	200 mW 30 mW 30 mW 30 mW	3 s 24 s 15 s 15 s	4 J/cm^2^	micro mass culture system (Pellet)	rMSCs	IR laser had highest stimulatory effect on chondrogenesis-related gene expression (Col 10)IR + blue laser increased the expression of SOX-9, Col 2, aggrecan.	Fekrazad et al., 2019 [82]
Investigating PBM effects associated with chitosan viscosupplementation for osteoarthritis	GaAlAs diode	808	50 mW	28 s	50 mJ/cm^2^	Chitosan hydrogel	OA in Wistar rats	PBM combined with chondrocyte-seeded hydrogel inhibited articular degeneration by boosting TGF-β, decreasing TNF-α, and enhancing Col 2 expression in vivo.	Tim et al., 2020 [83]
Assessment the effects of extrinsic PBM conjungated with cell adhesion pattern in inducing adipogenic differentiation of WJ-MSCs	NA	690	5 mW/cm^2^	10 s, every 2–3 days	50 mJ/cm^2^	Collagen microislands and TCPS	WJ-MSCs	The integration between EPM and collagen microislands exhibited the higher rate of adipogenic differentiation (~28%) than that of EPM and TCPS. A greater efficacy in inducing adipogenic differentiation of EPM and collagen microislands than using chemical factors was revealed.	Muneekaew et al., 2022 [80]
Evaluation of PBM therapy for chondrocyte-derived cellular responses in alginate scaffold	Infrared diode laser ((MLL-III-830 nm 2 W-EK10935)	830	0.1 W	32 s, every 2 days	5 J/cm^2^	Alginate scaffold	Chondrocyte	PBM (5 J/cm^2^) significantly upregulated cell proliferation, GAG synthesis, EV release, and SG formation in chondrocytes-seeded alginate scaffold.Multiple PBM sessions (one every 4 days) provided a greater improvement in chondrocyte-derived biological responses.	N.L.T. Hang et al., 2024 [18]
Determine GAG generation in differentiated MeSCs after PBM	LED (LH-SDT5W)	660	35.398 W/cm^2^	113 s 508 s	4 J/cm^2^ 18 J/cm^2^	Hydrogel	MeSCs	GAG production of PBM groups was significantly higher than in control group, but it was not between PBM groups.	Tong et al., 2024 [79]

Abbreviation: SSNPs, starch-stabilized magnetite nanoparticles; PBM, photobiomodulation; BMSCs, bone marrow mesenchymal stem cells; AC, articular cartilage; dECM, decellularized extracellular matrix; InGaAIP, indium-gallium-aluminum-phosphide; hDPSCs, human dental pulp stem cells; FBS, fetal bovine serum; rMSCs, rabbit mesenchymal stem cells; Col, collagen; SOX 9, sex-determining region Ybox 9; GaAlAs, gallium-aluminum-arsenide; TNF-α, tumor necrosis factor-alpha; TGF-β, transforming growth factor beta; WJ-MSCs, Wharton’s jelly mesenchymal stem cells; EPM, extrinsic photobiomodulation; TCPS, tissue culture polystyrene; EV, extracellular vesicle; SG, secretory granules; MeSCs, meniscus-derived stem cells.
